# Plasma cell myeloma lytic lesions mimicking vanishing bone syndrome in a young patient

**DOI:** 10.1259/bjrcr.20190025

**Published:** 2019-11-15

**Authors:** Margaret Mwania, Naushad Karim, Sarah Wambui, Shamshudin Mohammedali, Allan Njau

**Affiliations:** 1 Department of Radiology, Aga Khan University Hospital, Nairobi, Kenya; 2 Department of Orthopaedic Surgery, Aga Khan University Hospital, Nairobi, Kenya; 3 Department of Anatomic Pathology, Aga Khan University Hospital, Nairobi, Kenya

## Abstract

Plasma cell myeloma is a bone marrow disorder characterized by neoplastic proliferation of plasma cells within the bone marrow replacing normal cells. We present a case report of a 25-year-old female with bilateral lower and upper limb pains. She had been seen in various health facilities for the past 2 years with progressively worsening disability. Skeletal survey revealed multiple osteolytic lesions in the appendicular skeleton resembling vanishing bone syndrome. Ultrasound-guided biopsy was done with histological diagnosis of plasma cell myeloma. This case is unique because of the young age at presentation, HIV seropositive status and atypical appearance of the lesions.

Plasma cell myeloma tends to affect older people with up to 80% of patients diagnosed over the age of 65 years. Multiple myeloma is rare in those under 40 years old. Proposed risk factors include old age, male gender, African Americans, radioactivity, positive family history, occupational exposure (*e.g.* petroleum products), and obesity. It is a disorder characterized by neoplastic proliferation of plasma cells within the bone marrow replacing normal cells causing anemia and immunological alterations with bone destruction and fracture, bone pain, and hypercalcemia.^1^ Bone lysis is caused by myeloma cell-mediated promotion of osteoclast-mediated bony destruction and inhibition of osteoblast-mediated bone anabolism. Myeloma cells attach to osteoclasts directly through numerous adhesion molecules with resultant stimulation of osteoclastogenesis. Myeloma cells also secrete factors that inhibit differentiation of osteoblasts, such as Dickkopf 1 (DKK-1), tumour necrosis factor α (TNF-α), soluble frizzled-related protein-2 (SFRP-2), and Activin A. DKK-1 and SFRP-2 also inhibit the pathway for osteoblastic maturation.^2^ The typical manifestations of multiple myeloma can be summarized by the acronym, CRAB which represents, hypercalcemia, renal insufficiency, anemia, and bone lesions. Radiographically, the lesions could be in the form of a classic discrete lytic area (plasmacytoma), widespread osteopenia, or multiple lytic lesions affecting any part of skeleton, preferably spine, skull, and long bones. The classical presentation is multiple lytic lesions in the skull and spine. The higher the number of lesions, the poorer the prognosis. We report a case of multiple plasmacytomas resembling vanishing bone syndrome.

## Case report

The patient, 25-year-old female, HIV positive since birth and on antiretroviral medication, presented with a history of bilateral lower and upper limb pains with progressive lower limb weakness leading to inability to walk. She had been on wheelchair/immobile for 2 years and had been to multiple institutions where radiographs showed multiple lytic lesions but with no definitive diagnosis and management. She presented to Aga Khan University Hospital, Nairobi for second opinion. On physical examination, the patient was normotensive, pale, and had finger clubbing. She had multiple soft tissue swellings and deformity involving the left elbow, left dorsum of the hand, bilateral knees and above the right medial malleolus. Laboratory investigations revealed normocytic anemia with Hb of 5.6 g dl^−1^, MCV of 85.5fl, red blood cell count, 2.3 × 10^12^/l, white blood cell count 11.4 × 10^9^/l and platelet count 489 × 10^9^/l. The CRP was normal. Renal function tests were normal with creatinine levels of 86 µmol/l. The viral load was undetectable and the CD4 count was 733.

**Figure 1.**
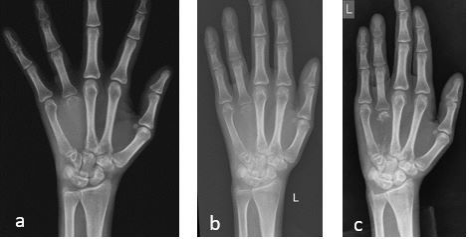
Left hand radiographs showing (a) lytic lesion with near complete destruction of the fourth metacarpal with progressive bone formation in (b) after 1 cycle of chemotherapy and (c) after 2 cycles of chemotherapy.

**Figure 2.**
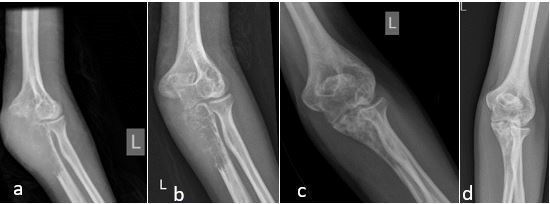
Left elbow radiographs showing (a) lytic lesion with soft tissue component in the proximal ulna with progressive bone formation in (b) after 1 cycle of chemotherapy (c) after 2 cycles of chemotherapy and (d) post radiotherapy and 4 cycles of chemotherapy.

**Figure 3.**
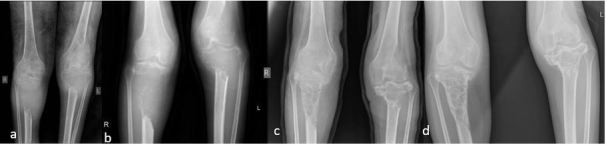
Knee radiographs showing (a) lytic lesions with soft tissue components in the bilateral proximal tibia and to a lesser extent the distal left femur with progressive bone formation in (b) after 1 cycle of chemotherapy (c) after 2 cycles of chemotherapy and (d) post-radiotherapy and 4 cycles of chemotherapy.

**Figure 4.**
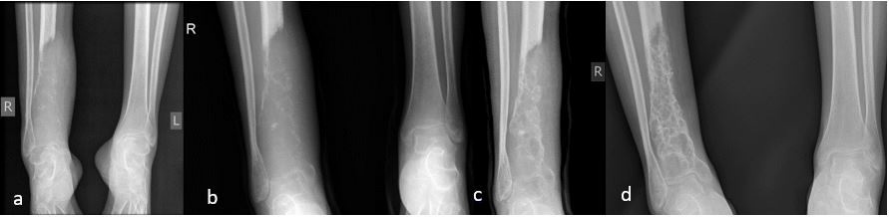
Right ankle radiographs showing (a) lytic lesion with soft tissue component in the distal right tibia with progressive bone formation in (b) after 1 cycle of chemotherapy (c) after 2 cycles of chemotherapy and (d) post radiotherapy and 4 cycles of chemotherapy.

**Figure 5.**
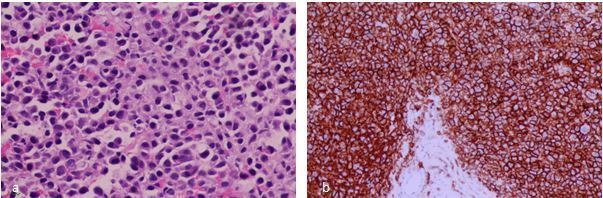
(a) Hematoxylin and Eosin; core biopsy of tibial lesion, sheets of plasmacytoid cells exhibiting eccentric nuclei, eosinophilic cytoplasm and clock face chromatin pattern (400 × magnification). (b) Immunohistochemistry; neoplastic cells show strong diffuse membrane positivity for CD 138 (200 × magnification)

In view of the multiple sites of bone pain and deformity, skeletal survey was ordered which revealed multifocal lytic expansile lesions with gross bony destruction and soft tissue swelling. The lesions resembled vanishing bone syndrome (Gorham’s disease) as shown in Figures 1a–4a below. Ultrasound-guided biopsy of the proximal leg soft tissue swelling revealed sheets of plasmacytoid cells in keeping with plasma cell myeloma as shown in Figure 5 below. The patient was referred to a hematologist and further work-up was done. Serum electrophoresis showed monoclonal gammopathy of IgG κ. Serum free light chain showed both κ and λ chains were elevated with κ 991 (3.3–19.4) mg l^−1^ and λ 43 replace_with (43 mg/l^−1^) (5.7–26.3). Serum Beta-2 Microglobulin was also elevated at 16269 ng ml^−1^ (670–2143). She was subsequently started on 28 day cycle, bortezomib, cyclophosphamide, dexamethasone and analgesics on a weekly basis. Immobilization by bilateral above knee casts was also done with her pain subsiding and was discharged for follow up in the outpatient clinic. Follow-up skeletal survey after one cycle, two cycles and after four out of six cycles of chemotherapy and completion of radiotherapy showed progressive new bone formation in the affected areas with interval resolution of the soft tissue components. See Figures 1–4 below. No new lesion was seen.

## Discussion

Plasma cell myeloma tends to affect older people over 65 years old of age. It is rare in those under 40 years old. In the young like in our patient, it usually has an atypical clinical presentation with multiple or solitary extramedullary plasmacytoma and osteolytic lesions. The clinical behavior of multiple myeloma in adolescents and young adults has been suggested to be more indolent with a more favorable prognosis.^3^ This may explain the good response to treatment for our patient after chemotherapy and radiotherapy.

HIV-infected patients have increased risks for plasma cell disorders. The exact mechanisms for the increased risk of plasma cell disorders in HIV patients remain poorly understood. Plasma cell myeloma in HIV/AIDS patients has several unique characteristics; occur at younger age, like in this case; shows atypical clinical evolution, atypical histopathological findings, progression is rapid and overall survival short^4^ We however can postulate that the relatively slow progression in our patient before the diagnosis was made and good treatment response after the diagnosis may be due to good compliance of ARVS with undetectable viral load and good CD four count.

Any bone can be affected in multiple myeloma and the most common cited areas in literature are the spine (49%), skull (35%), pelvis (34%), ribs (33%), humeri (22%), femora (13%) and mandible (10%)^5^ . However, in our case, the appendicular skeleton was affected with no radiographic evidence of disease in the ribs and spine.

Gorham’s disease was considered a differential on the skeletal survey done at our institution due to progressive and profound osteolysis and lack of periosteal reaction. It is a rare and poorly understood bone disease of uncertain etiology. It is thought to be non-hereditary and found to occur most commonly in young adults. The osteolysis is thought to occur due to an increased number of stimulated osteoclasts likely secondary to non-neoplastic vascular and lymphatic proliferation. Imaging features include profound osteolysis and bone resorption without any compensatory periosteal reaction or surrounding sclerotic reaction.

The other differential to consider is osteolytic metastasis. Metastatic disease is however rarely seen in the distal appendicular skeleton, especially with sparing of the axial skeleton. In our case, the patient was also not known to have any known malignancy at the time of presentation.

Involvement of multidisciplinary team is important because these findings should have led to suspicion of myeloma and this case should increase awareness of the unique features of plasma cell myeloma in HIV infected patients.

## Learning points

- Plasma cell myeloma tends to affect older people and is rare in those under 40 years old, however, it should be considered in younger patients with multiple lytic lesions.
- In the young, plasma cell myeloma usually has an atypical clinical presentation with multiple or solitary extra medullary plasmacytoma and osteolytic lesions and an indolent as well as favorable prognosis.
- HIV-infected patients are at an increased risk for plasma cell disorders and these tend to occur at an earlier age with atypical clinical presentation.
- Patients with HIV tend to have a rapidly progressive disease with short survival, however, we postulate that those on treatment with good CD4 counts and low viral loads may have similar disease progression to HIV negative patients
